# Combined Effect of Walking and Forest Environment on Salivary Cortisol Concentration

**DOI:** 10.3389/fpubh.2019.00376

**Published:** 2019-12-12

**Authors:** Hiromitsu Kobayashi, Chorong Song, Harumi Ikei, Bum-Jin Park, Takahide Kagawa, Yoshifumi Miyazaki

**Affiliations:** ^1^Department of Nursing, Ishikawa Prefectural Nursing University, Kahoku, Japan; ^2^Center for Environment, Health and Field Sciences, Chiba University, Chiba, Japan; ^3^Department of Forest Resources, Kongju National University, Yesan-gun, South Korea; ^4^Forestry and Forest Products Research Institute, Tsukuba, Japan; ^5^Department of Environment and Forest Resources, Chungnam National University, Daejeon, South Korea

**Keywords:** shinrin-yoku, forest therapy, walking, salivary cortisol, interaction

## Abstract

We investigated the effects of walking in a forest environment on salivary cortisol concentrations. Seventy-four young male participants walked for 15 min in forested and urban environments, and saliva was collected before and after walking. Our previous study reported salivary cortisol concentrations after walking only. This study was aimed at clarifying the combined effects of walking and environment by comparing post-walking data with pre-walking data. Walking in a forest environment decreased mean cortisol concentration from 9.70 to 8.37 nmol/L, whereas walking in an urban environment barely changed mean cortisol concentration, from 10.28 to 10.01 nmol/L. Two-way repeated analysis of variance revealed a significant interaction effect between the environment and walking (*p* < 0.001) in addition to the main effects of each (*p* < 0.001 and *p* = 0.001, for walking and environment, respectively). For further analysis, the proportion of participants who exhibited decreased cortisol after forest-walking was compared with the previously reported proportion of participants who exhibited decreased cortisol after viewing forest landscapes. Although the proportion of positive responders was slightly higher after walking (69%) than it was after viewing (60%), this difference was not statistically significant (*p* = 0.093). The present study revealed a significant combined effect of walking and the environment on cortisol concentrations.

## Introduction

Recent years have seen growing interest in the therapeutic effects of forest environments on human health. “Shinrin-yoku,” a Japanese term that means “forest-bathing,” is now used internationally to refer to forest exposure for therapeutic or preventive health purposes (1–4). As Bowler et al. (5) reported, 13 studies on the effects of walking in natural environments were published between 1991 and 2008, most of which reported that nature therapy decreased negative emotions, such as anger, fatigue, and sadness, and enhanced positive emotions, such as energy. The psychological benefits of forest-walking have been supported by many subsequent studies (6, 7). However, the benefits of forest-walking are not only psychological. Other studies have shown that it has desirable effects on physiological functions as well, including decreased blood pressure (8–11), increased high-frequency component (HF) of heart rate variability (HRV) (12–15), decreased cerebral activity (16), and enhanced immune functions (17, 18).

Cortisol level is another metric that has been used in studies on the therapeutic effects of forest exposure (16, 19–24). Cortisol is a steroid hormone produced by the zona fasciculata of the adrenal cortex to regulate carbohydrate, fat, and protein metabolism. Cortisol level is considered to be an indicator of the activity of the hypothalamic-pituitary-adrenal (HPA) axis, which is responsible for corticotropin-releasing hormone (CRH) and adrenocorticotropin (ACTH) secretion. The HPA system is among the major stress response systems in humans. ACTH-mediated cortisol secretion increases blood pressure and glucose levels while suppressing the immune system; this set of physiological reactions is called the “fight-or-flight” response. Cortisol level has accordingly been used as a biological indicator of stress.

The authors have previously reported that walking in a forested environment significantly reduced salivary cortisol concentrations in 74 young male participants (25). In that study, post-walking data were compared between subjects who had walked in forest environments and subjects who had walked in urban environments, but pre-walking data was not included in the analysis. Given that salivary cortisol is affected not only by environmental stress but also by physical exercise such as walking, however, it is desirable to separate the effect of walking and that of environment in studies of nature therapy. Several previous studies have reported that walking in a forested environment reduces serum (9, 19) or salivary (20, 21, 23) cortisol concentrations; however, no study has yet clearly demonstrated the interaction between the effects of walking and environment.

In this study, salivary cortisol data collected before walking was analyzed along with our previously published results. Two-way analysis of variance (ANOVA) was performed to examine the combined effects of walking and forest exposure. In addition, we compared the results of forest-walking with those of forest-viewing reported in our previous study (24) to determine whether forest-walking has a stronger beneficial effect than forest-viewing has.

## Materials and Methods

### Study Sites and Participants

The study areas were seven forests and seven urban areas across Japan. Urban areas were selected near city centers or railway stations. Seventy-five Japanese male university students (aged 20–29 years) participated in the experiments. As one participant's pre-walking cortisol concentration data was missing, however, salivary cortisol concentrations of 74 participants were analyzed.

Demographic parameters of the 74 male participants are shown in Table 1. According to the Japanese National Health and Nutrition Survey 2016 (26), mean height and weight of Japanese males aged 20–29 years were reported as 171.5 cm and 67.6 kg, respectively. These reference values were almost identical to those of our participants. Thus, the participants in this study can be regarded as a representative sample of the Japanese young male population.

**Table 1:** Demographics of the male participants (*n* = 74).

	**Age (year)**	**Height (cm)**	**Weight (kg)**	**BMI**
Mean	22.4	172.4	65.4	22.0
SD	1.8	5.8	10.3	3.1
Max	29	187.5	110.0	33.6
Min	20	155.0	50.0	17.4

*BMI, Body mass index*.

None of the participants reported a history of physical or psychiatric disorders. During the study period, alcohol and tobacco consumption was prohibited and caffeine consumption was limited.

### Experimental Design

The experiment was performed over two consecutive days at each of the seven study areas. Prior to the experiment, the aim of this study and the experimental protocol were explained and general instructions were provided to the participants. After this orientation, the participants visited and previewed the two experimental sites in their study area. The participants in each study area were randomly divided into two groups, one of which performed the experiment in the forest area prior to the urban area, while the other group performed the experiment in the urban area prior to the forest area.

On the experiment day, participants woke up between 6:30 and 7:30 a.m. After arriving at experimental site, the participants rested in a chair for 5 min and then saliva was sampled before they began walking. Each participant walked in his assigned urban or forested environments for 15 min. Walking paths were nearly level in all environments. In this study, participants were instructed to walk relatively slowly and to walk at the same speed in both the environments. We confirmed that no significant difference was observed regarding walking speed between forest and urban environments. Second saliva sample was collected immediately after the walking.

After this first experiment, all participants spent the night in identical single rooms at the same hotel. On the second day, the participants switched field sites. The experimental protocol for the second day was the same as that for the first day.

### Salivary Cortisol Measurements

Salivary samples were collected before lunch. The time of saliva collection varied from 9:30 a.m. to 12:00 p.m. Although the time of saliva collection varied among the participants, each participant was measured at approximately the same time on the two experimental days.

Saliva was collected using the Salivette® system (No. 51.1534; Sarstedt, Nümbrecht, Germany). Each participant held two pieces of absorbent cotton in his mouth for 2 min, and the saliva in these cotton wads was later extracted by centrifugation. The samples were immediately frozen and transported to the laboratories of SRL, Inc., (Tokyo, Japan). Each sample consisted of a 0.25-mL aliquot of saliva, and its cortisol concentration was analyzed by radioimmunoassay.

In addition to salivary cortisol, heart rate, blood pressure, salivary immunoglobulin A concentration, and Profile of Mood States (POMS) were also measured in this experiment; the present study, however, deals only with the salivary cortisol results. Post-walking data for the other physiological metrics were reported in our previous paper (25).

### Statistical Analysis

The combined effect of environment (urban or forest) and walking (before or after) on salivary cortisol concentration was statistically tested by two-way repeated measure analysis of variance (ANOVA). Both environment and walking were analyzed as within-subject factors. In addition to the *p*-value, partial eta-squared (η^2^) was calculated as an effect size for the factor. The simple main effect was then analyzed as a *post-hoc* test. The above statistical tests were performed on natural-log-transformed data rather than raw cortisol concentrations because cortisol concentrations exhibit a non-normal distribution except in the early morning (27, 28).

For further analysis, the proportions of participants who exhibited positive and negative responses were compared. Positive response was defined as having a lower cortisol concentration immediately before walking in the forest environments than immediately before walking in the urban environments. Negative response was defined as the opposite. The results were compared with those of our previous study (24) in which we investigated salivary cortisol concentrations in 348 young male participants after they viewed forest landscapes. The difference between the present and previous studies with respect to the ratio of negative to positive responders was compared by the Chi-squared test. A *p* < 0.05 was considered to indicate statistical significance for the ANOVA and Chi-squared tests. These statistical analyses were performed using R ver. 3.5.3.

## Results

Statistics for cortisol concentration are presented in Table 2. Note that the post-walking cortisol concentrations are the data reported in our previous paper (25).

**Table 2:** Statistics for salivary cortisol concentrations in forest and urban environments (*n* = 74).

	**Salivary cortisol concentration (nmol/L)**			
	**Urban**		**Forest**	
	**Before**	**After**	**Before**	**After**
Mean	10.28	10.01	9.70	8.37
SD	4.58	3.98	4.04	2.90
Median	8.83	8.97	8.69	7.73
Skewness^*^	0.30	0.14	0.24	−0.13
Kurtosis^*^	−0.39	−0.46	0.41	0.21

* *Skewness and kurtosis were calculated based on logarithmic-transformed cortisol concentration data*.

Walking in a forest environment decreased mean cortisol concentration from 9.70 to 8.37 nmol/L. Walking in an urban environment, on the other hand, barely changed mean cortisol concentration from 10.28 to 10.01 nmol/L. Similar tendencies were also observed in the median values, which changed from 8.69 to 7.73 nmol/L for forest-walking, and from 8.83 to 8.97 nmol/L for urban-walking.

The skewness and kurtosis presented in Table 2 were calculated based on log-transformed cortisol concentrations whereas the other statistics were calculated based on raw cortisol concentrations. The skewness and kurtosis values were nearly zero, suggesting that the log-transformed cortisol concentrations indicated almost normal distribution.

The results of ANOVA are summarized in Table 3. Both environment (urban or forest) and walking (before or after) were statistically significant factors (*p* = 0.001 and *p* < 0.001, respectively). In addition to the main effects, the interaction effect between environment and walking was statistically significant (*p* < 0.001).

**Table 3:** Results of two-way repeated measure ANOVA (*n* = 74).

**Factor**	***F*_**(1, 73)**_**	***p***	**Partial η^2^**
Walking (before/after)	12.59	<0.001	0.147
Environment (urban/forest)	10.97	0.001	0.131
Interaction (environment × walking)	15.17	<0.001	0.172

*ANOVA (analysis of variance) was performed on log-transformed cortisol concentration data*.

**Figure 1:**
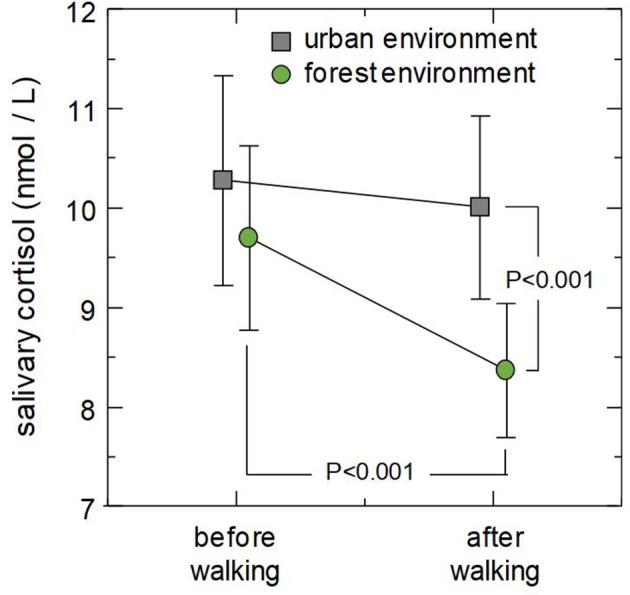
Combined effect of environment and walking on salivary cortisol concentrations. Markers indicate mean cortisol concentrations in urban (square) and forest (circle) environments and error bars designate ±2SE (standard error). *P*-values are the results of *post-hoc* analysis (simple main effect test).

The results of the *post-hoc* comparisons are shown in Figure 1. The *post-hoc* tests revealed that mean cortisol concentration after walking was significantly lower in forest environments than in urban environments (*p* < 0.001), whereas mean cortisol concentration before walking was not significantly different between forest and urban environments (*p* = 0.207). Comparison of the data taken before and after walking revealed that walking in a forest environment significantly decreased salivary cortisol (*p* < 0.001), whereas walking in an urban environment did not significantly change salivary cortisol (*p* = 0.658).

Fifty-one (69%) of the 74 participants exhibited a positive response (i.e., a decrease in salivary cortisol) to walking in a forest environment. The remaining participants exhibited a negative response (*n* = 16, 22%) or unchanged cortisol levels (*n* = 7, 9%). A comparison between the ratio of positive to negative responders in the present walking study and that in our previous viewing study (24) is summarized in Table 4. In the previous viewing study, 209 (60%) of 348 participants were positive responders while the remaining participants exhibited a negative response (*n* = 118, 34%) or unchanged cortisol levels (*n* = 21, 6%). A Chi-square test revealed that the difference in the distribution of positive and negative responders between the walking and viewing studies was not statistically significant (*p* = 0.093), although the percentage of positive responders was considerably larger in the walking study than in the viewing study (24).

**Table 4:** Number of participants who exhibited positive/negative responses in salivary cortisol after forest exposure.

	**Negative response**	**Unchanged**	**Positive response**
Walking (*n* = 74)	16 (22%)	7 (9%)	51 (69%)
Viewing (*n* = 348)^*^	118 (34%)	21 (6%)	209 (60%)
Chi squared = 4.76, *p* = 0.093			

* *Results on cortisol levels after viewing urban or forest landscapes were presented in our previous report (24)*.

## Discussion

### Effect of Walking in a Forest Environment on Salivary Cortisol Concentration

This study aimed to investigate and to distinguish between the effect of walking and that of forest exposure on salivary cortisol concentration. Walking in a forest environment significantly reduced salivary cortisol concentrations, whereas walking in an urban area had little effect on cortisol. Moreover, cortisol concentrations before walking were not significantly different between the forest and urban environments, whereas cortisol concentrations after walking were significantly lower in the forest environments than in the urban environments. In short, an interaction effect between walking and environment was clearly demonstrated in this study.

In contrast with the present results, several previous studies have reported finding no cortisol-reducing effect of forest-walking. In a study on the effects of walking for 45 min along a mountain path (29), salivary cortisol was repeatedly measured before, immediately after, 20 min after and 40 min after each participant's walk but did not significantly vary with time. In a study conducted in Finland (22), subjects were exposed to three different environments (forest, park and urban). Their exposure to these environments consisted of 15 min of viewing and 30 min of walking. An ANOVA on salivary cortisol revealed that only time factor (before and after exposure) was significant, whereas environment type and interaction effect were insignificant. Komori et al. (30) investigated the effects of walking in urban and forest environments on salivary cortisol concentration and found that, as in the present study, walking in a forest environment significantly reduced cortisol concentration, yet urban-walking also reduced cortisol concentration considerably but not significantly, and cortisol concentrations after walking were not significantly different between forest and urban environments. In short, the interaction between walking and environment was unclear in their results. Accordingly, the significant interaction demonstrated in the present study is considered to be a novel finding in the field of nature therapy.

These other studies' negative results regarding the interaction between walking and forest exposure might be attributed to the high intensity of walking as an exercise. Physical exercise itself is known to increase cortisol concentrations: for example, Cook et al. (31) reported an extremely high cortisol concentration (100 nmol/L) in a marathon runner just after completion of the race. Jacks et al. (32) also reported higher salivary cortisol levels after 1 h of high-intensity exercise (76% VO_2_max); after less than 20 min of exercise, on the other hand, salivary cortisol concentrations were not affected regardless of the intensity. To achieve an observable increase in salivary cortisol, therefore, both high exercise intensity (more than 70% VO_2_max) and an exercise duration of at least 20 min might be required (33). As some of the forest-walking experiments in previous studies were more intense and longer in duration than our own, the walking in these experiments may have increased subjects' cortisol concentrations, which would obscure the cortisol-reducing effect of the forest environment.

Longer walking duration is linked to another difficulty related to the interpretation of cortisol responses. It is well known that cortisol exhibits clear diurnal variation. Cortisol shows a maximum concentration at 30–40 min after waking and then declines throughout the day. This phenomenon is known as the cortisol awakening response (CAR) (34). Therefore, if two measurements of salivary cortisol are taken more than an hour or two apart and then compared, the second measurement will be lower than the first simply due to the passage of time. Taking all of these points into account, we consider that high-intensity walking for long periods (e.g., mountain climbing) is an inappropriate experimental design for studying the effects of forest-walking on cortisol levels.

### Forest-Walking vs. Forest-Viewing

The biophilia hypothesis was proposed by Edward O. Wilson in 1984 (35). Biophilia is defined as the “innate tendency to focus on life and life-like processes”(36). In primitive environment, natural features, including trees or forests, provided food, water, or shelter to our ancestors. Thus, biophilia may have been an adaptive characteristic. However, certain people strongly dislike natural settings. This tendency is called biophobia (37). Considering that various types of dangers also exist in the natural environment (e.g., predators and poisonous animals), biophobia is additionally an adaptive psychological trait. Therefore, the effect of the natural environment on humans is dual-faced. Hence, we considered that exploring both the positive and negative aspects of the natural environment is important.

In another of our previous studies (38), the effect of walking in a forest environment and the effect of viewing a forest landscape on HRV indices was investigated. In the study, a positive responder was defined as a participant who exhibited an increase in log-transformed high-frequency component (lnHF). Regarding the lnHF of HRV, the proportion of positive responders was ~65% in the walking study and ~79% in the viewing study; this difference is statistically significant (*p* < 0.01).

A similar analysis was conducted on salivary cortisol concentrations. In the present results, ~69% of the participants exhibited a positive response to forest-walking. In our previous study on the effect of viewing a forest landscape (24), the proportion of positive responders was ~60%. Contrary to the lnHF of HRV, with regard to cortisol levels, the proportion of positive responders was considerably higher in the walking study than in the viewing study, though this difference did not reach significance. One possible reason for this result might have been an insufficient sample size. The sample size in the viewing study was 348, while the sample size in the walking study was only 74; thus the statistical power might have been insufficient to detect the difference. Therefore, we cannot conclude from this result that there is no difference between the effects of forest-walking and those of forest-viewing on salivary cortisol concentrations.

Antonelli et al. (3) performed a meta-analysis on the effects of forest exposure on salivary cortisol concentrations. They analyzed six studies on forest-viewing (including our previous results) and four studies on forest-walking using a “forest plot” to represent their results. The effect of forest-viewing (i.e., the mean difference in post-viewing cortisol levels between forest and urban environments) was estimated at −0.05 μg/dl with a 95% confidence interval (CI) of −0.07 to −0.04 μg/dl. The effect of forest-walking, on the other hand, was estimated at −0.04 μg/dl with a 95% CI of −0.07 to −0.01 μg/dl. In short, they found that forest-walking had a slightly smaller effect on salivary cortisol than forest-viewing had. This finding is discrepant with the results of this study. At present, we cannot clearly conclude that either viewing or walking is more effective at reducing salivary cortisol concentrations. Further investigations will be needed on the difference between viewing and walking regarding the therapeutic effect of the forest environment.

## Conclusion

The present study explored the interaction effect between walking (before and after) and environment (forest and urban) on salivary cortisol concentrations, although previous studies have reported that this interaction is unclear. Furthermore, a comparison of walking outcomes with viewing outcomes showed that the proportion of positive responders was somewhat larger after walking than after viewing, although this difference was not significant. It therefore remains unclear whether forest-walking is more therapeutic than viewing a forest landscape. This is an important question in the field of forest therapy, and future meta-analyses on cortisol are expected to answer it.

## Data Availability Statement

The datasets generated for this study are available on request to the corresponding author.

## Ethics Statement

The study was conducted in accordance with the Declaration of Helsinki, and the protocol was approved by the Ethics Committee of the Forestry and Forest Products Research Institute, Japan (project identification code number: 16-558), and the Center for Environment, Health and Field Sciences, Chiba University, Japan (project identification code number: 5). Participants were informed about the purposes and procedures of the study and provided written informed consent prior to enrollment. They were free not to attend or to cease participation in the program at any time.

## Author Contributions

HK contributed to statistical analysis, interpretation of the results, and manuscript preparation. CS and HI were involved with data acquisition and initial analysis of the results. B-JP and TK participated in data acquisition and study design. YM had an important role in the research, particularly in experimental design, data acquisition, and manuscript preparation. All authors contributed to the preparation of the manuscript and are responsible for the final editing and approval.

### Conflict of Interest

The authors declare that the research was conducted in the absence of any commercial or financial relationships that could be construed as a potential conflict of interest.
